# Synthesis and Biological Evaluation of 5-Fluoro-2-Oxindole Derivatives as Potential α-Glucosidase Inhibitors

**DOI:** 10.3389/fchem.2022.928295

**Published:** 2022-06-23

**Authors:** Jing Lin, Qi-Ming Liang, Yuan-Na Ye, Di Xiao, Li Lu, Meng-Yue Li, Jian-Ping Li, Yu-Fei Zhang, Zhuang Xiong, Na Feng, Chen Li

**Affiliations:** School of Biotechnology and Health Sciences, Wuyi University, Jiangmen, China

**Keywords:** oxindole, α-glucosidase, inhibition, docking, kinetics

## Abstract

α-Glucosidase inhibitors are known to prevent the digestion of carbohydrates and reduce the impact of carbohydrates on blood glucose. To develop novel α-glucosidase inhibitors, a series of 5-fluoro-2-oxindole derivatives (**3a** ∼ **3v**) were synthesized, and their α-glucosidase inhibitory activities were investigated. Biological assessment results showed that most synthesized compounds presented potential inhibition on α-glucosidase. Among them, compounds **3d**, **3f**, and **3i** exhibited much better inhibitory activity with IC_50_ values of 49.89 ± 1.16 μM, 35.83 ± 0.98 μM, and 56.87 ± 0.42 μM, respectively, which were about 10 ∼ 15 folds higher than acarbose (IC_50_ = 569.43 ± 43.72 μM). A kinetic mechanism study revealed that compounds **3d**, **3f**, and **3i** inhibited the α-glucosidase in a reversible and mixed manner. Molecular docking was carried out to simulate the affinity between the compound and α-glucosidase.

## 1 Introduction

Diabetes is a chronic metabolic disorder disease that increases the risk of cancer, stroke, peripheral arterial disease, cardiovascular disease, retinopathy, and kidney disease. (Wang et al., 2017; Sonia et al., 2019; Proença et al., 2019; Proença et al., 2017; Proença et al., 2018; Rocha et al., 2019; Santos et al., 2018; Wu et al., 2014; Wu et al., 2017). The prevalence of diabetes at all ages worldwide is rising. It is estimated that by 2030, the prevalence of diabetes may rise from 2.8% (171 million) in 2000 to 4.4% (366 million) (Zhong et al., 2019). Type 2 diabetes, which is characterized by insulin resistance, is the most common, which accounts for approximately 90% of all diabetic patients (Taha et al., 2015; Leong et al., 2019; Settypalli et al., 2019).

α-Glucosidase is an indispensable enzyme in the sugar metabolism pathway of organisms, and its main function is to hydrolyze glycosidic bonds into glucose (Chaudhry et al., 2019; Dan et al., 2019; Gollapalli et al., 2019; Krishna et al., 2019; Mendieta-Moctezuma et al., 2019; Spasov et al., 2019; Ye et al., 2019). Thus inhibiting the α-glucosidase would obviously control the postprandial hyperglycemia. α-Glucosidase inhibitors can block the hydrolysis of 1, 4-glycosidic bonds and delay the hydrolysis of carbohydrates into glucose, resulting in the effective reduction of postprandial blood sugar (Al-Salahi, et al., 2018; Qamar, et al., 2018; Shah, et al., 2018; Wang, et al., 2018). Up to now, a great number of naturally occurring and synthetic α-glucosidase inhibitors have been reported. However, only several well-known inhibitors, such as acarbose, voglibose, and miglitol, are used clinically as first-line drugs. Moreover, these drugs have uncomfortable side effects (e.g., flatulence, abdominal pain, and diarrhea) (Taha et al., 2018a; Kasturi et al., 2018; Prachumart et al., 2018). These prompt us to develop effective and safe α-glucosidase inhibitors from natural sources.

Lots of compounds from natural sources have shown potential inhibitory activity on α-glucosidase. Oxindoles, the important indole-based derivatives, widely exist in many natural alkaloids. It was reported that oxindoles have the ability to inhibit the α-glucosidase (Khan et al., 2014; Asadollahi-Baboli and Dehnavi, 2018; Taha et al., 2018b). Moreover, oxindoles have attracted much attention due to their broad-spectrum biological activity, such as anti-inflammatory, anti-bacterial, and anti-tumor. (Yang et al., 2014; Xu et al., 2016; Alvarez et al., 2018; Bao et al., 2018; Huang et al., 2019). In addition, fluorine, a key atom in medicine, might enhance metabolic stability, improve the pharmacodynamic effect, and eliminate active metabolic intermediates (Johnson et al., 2020). Hence, 5-fluoro-2-oxindole was selected as the leading structure to synthesize the title compounds (**3a** ∼ **3u**) through the condensation with the substituted aromatic aldehydes, followed by the screening on α-glucosidase inhibitory activities and the molecular docking studies.

## 2 Results and Discussion

### 2.1 Chemistry

**SCHEME 1:**
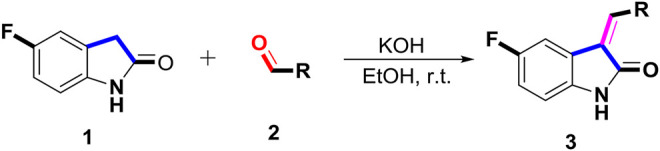
Synthetic route to 5-fluoro-2-oxindole derivatives (**3a ∼ 3u**). Reagents and conditions: 5-fluoro-2-oxindole (1.0 mmol, 1.0 equiv), substituted aldehydes (1.5 mmol, 1.5 equiv), KOH (6.0 mmol, 6.0 equiv), and EtOH, r. t., 3 h.

The 5-fluoro-2-oxindole derivatives (**3a ∼ 3v**) were prepared according to the synthetic route shown in Scheme 1. As the starting material, 5-fluoro-2-oxindole (**1**) was condensed with the substituted aromatic aldehydes (**2a ∼ 2v**) in the presence of KOH to produce the title compounds (**3a ∼ 3v**). The structures of compounds **3a ∼ 3v** were characterized by ^1^H NMR, MS, and melting point.

### 2.2 α-Glucosidase Inhibition Assay

**FIGURE 1:**
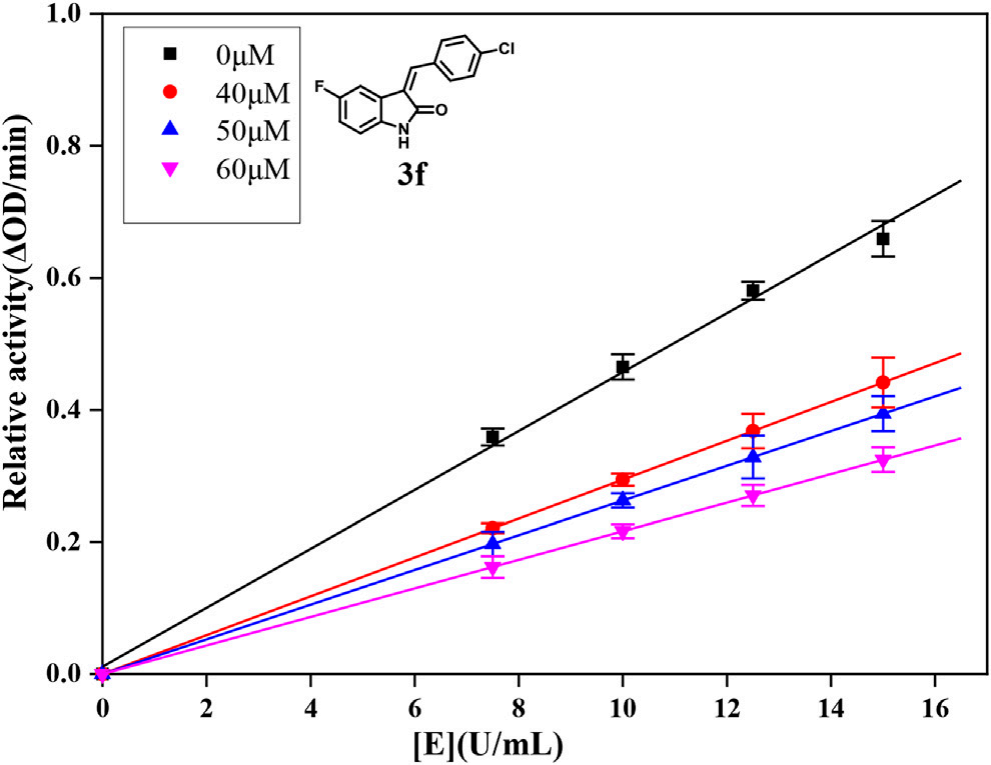
Inhibition mechanism determination of compound **3f** on α-glucosidase.

α-Glucosidase from *Saccharomyces cerevisiae* (EC 3.2.1.20) was widely accepted and used to evaluate the inhibitory activity against α-glucosidase. Then, the inhibitory activity of compounds **(3a ∼ 3v**) on α-glucosidase from *S*. *cerevisiae* was investigated using *p*-NPG as the substrate. First, the inhibitory activities of compounds (**3a ∼ 3v**) were screened at a concentration of 100 μM. As shown in Table 1, compounds **3d**, **3f**, and **3i** presented better activities, with inhibition of ∼90% at a concentration of 100 μM and those of compounds (**3g**, **3n**, **3p**, and **3r**) were ∼50% at a concentration of 100 μM, while those of other compounds were below 50% at a concentration of 100 μM. Then, IC_50_ values of compounds **3d**, **3f**, **3i**, **3n**, **3p**, and **3r** were measured due to their better inhibitory activities. The IC_50_ values are summarized in Table 1, and the inhibitory activities of compounds **3d**, **3f**, and **3i** on α-glucosidase are presented in Figure 1. For analyzing the inhibitory activities of compounds (**3a** ∼ **3v**), the inhibitory activities of 5-fluoro-2-oxindole and acarbose were investigated. Among all compounds, compounds **3d**, **3f**, and **3i** exhibited much better potent inhibitory activity with IC_50_ values of 56.87 ± 0.42 μM, 49.89 ± 1.16 μM, and 35.83 ± 0.98 μM, respectively, which were about 10 ∼ 15 folds higher than that of acarbose (IC_50_ = 569.43 ± 43.72 μM) and significantly better than that of 5-fluoro-2-oxindole (IC_50_ = (7.51 ± 0.17)×10^3^ μM).

**TABLE 1:** α-Glucosidase inhibitory activities of compounds **(3a ∼ 3v**).

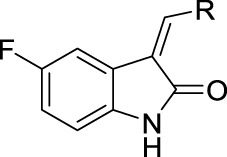			
**Compound**	**R**	**Inhibition rate at a concentration of 100 μM (%)**	**IC_50_ (μM)**
**3a**	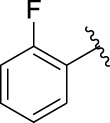	7.29 ± 0.16	>100^a^
**3b**	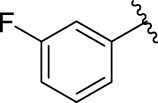	5.19 ± 0.79	>100^a^
**3c**	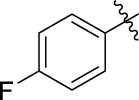	9.55 ± 0.13	>100^a^
**3d**	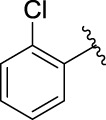	89.19 ± 0.14	49.89 ± 1.16
**3e**	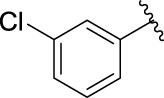	21.64 ± 0.78	>100^a^
**3f**	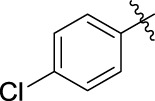	90.52 ± 0.27	35.83 ± 0.98
**3g**	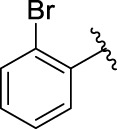	53.71 ± 0.47	95.68 ± 0.28
**3h**	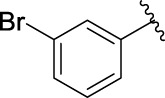	31.61 ± 0.21	>100^a^
**3i**	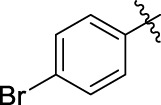	92.86 ± 0.32	56.87 ± 0.42
**3j**	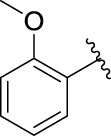	19.17 ± 1.21	>100^a^
**3k**	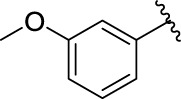	27.25 ± 1.47	>100^a^
**3l**	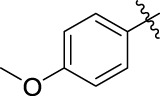	15.70 ± 0.71	>100^a^
**3m**	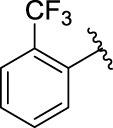	8.43 ± 1.14	>100^a^
**3n**	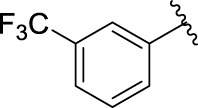	52.79 ± 1.68	96.78 ± 0.72
**3o**	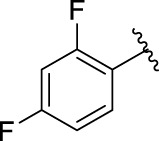	18.78 ± 1.15	>100^a^
**3p**	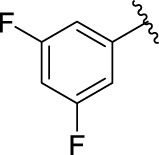	55.89 ± 1.71	92.62 ± 0.45
**3q**	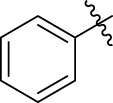	10.01 ± 1.75	>100^a^
**3r**	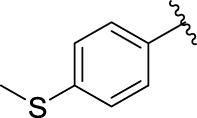	60.8 ± 1.27	90.56 ± 1.87
**3s**	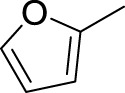	4.49 ± 1.88	>100^a^
**3t**	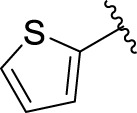	5.67 ± 1.11	>100^a^
**3u**	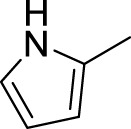	3.77 ± 1.35	>100^a^
**3v**	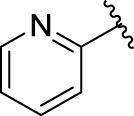	3.99 ± 1.28	>100^a^
**5-Fluoro-2-oxindole**			(7.51 ± 0.17) × 10^3^
**Acarbose**			569.43 ± 43.72

a The inhibitory activity of test compounds at 100 μM is less than 50%.

### 2.3 Structure–Activity Relationships

Then, the structure–activity relationships of compounds (**3a ∼ 3v**) were analyzed according to the experimental data in Table 1. First, the steric effect of substituents at aldehydes was investigated based on the inhibitory activities of compounds **3a/3b/3c**, **3d/3e/3f**, **3g/3h/3i**, and **3m/3n**, with -F, -Cl, -Br, and -CF_3_ at ortho-, meta- and para-positions of the benzene ring, and the order of the inhibitory activities is 4- > 2- > 3-. When the substituent was OCH_3_ (**3j/3k/3l**), it turned out just the opposite. Second, the electronic effect of substituents was considered. The introduction of -F, -Cl, -Br, -CF_3_, and -OCH_3_ at phenyl para-position (compounds **3c**, **3f**, **3i**, **3l**, and **3r**) could enhance the inhibitory activities with the inhibitory activity order of -Cl > -Br > -SCH_3_ > -OCH_3_ > -F. It could be seen that the inhibitory activity has no correlation with the steric and electronic effects of substituents at aldehydes. Furthermore, the introduction of various heterocycles (compounds **3s**, **3t**, **3u**, and **3v**) presented a negative effect on inhibitory activity. It could be concluded that the introduction of the substituents at the benzyl para-position of substituted aldehydes is beneficial to the improvement of the inhibitory activity. Therefore, the further derivatization of title compounds might be focused on the screening of substituents at the benzyl para-position of substituted aldehydes.

### 2.4 Inhibitory Mechanism Analysis

**FIGURE 2:**
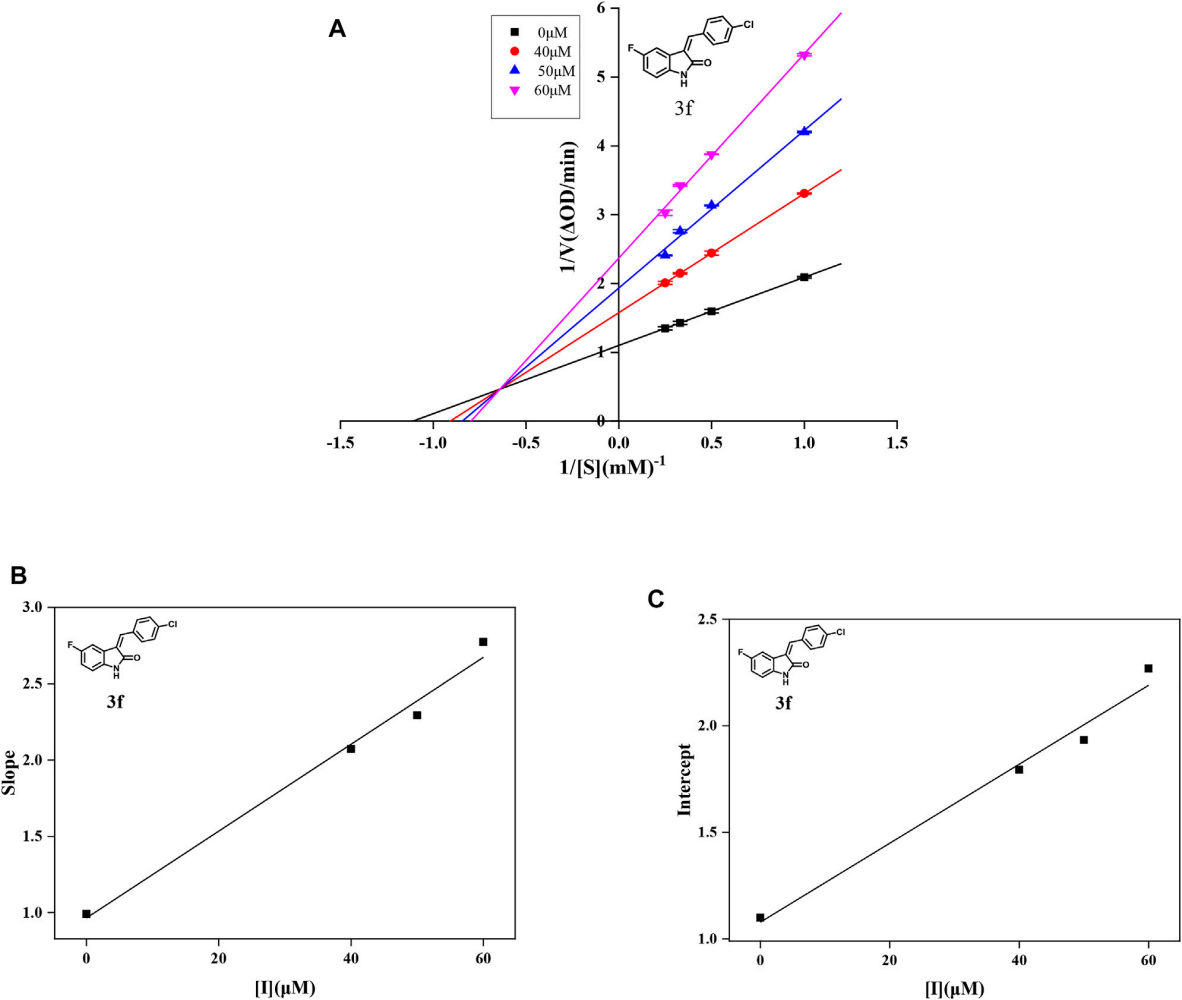
**(A)** Lineweaver–Burk plots of compound **3f** on α-glucosidase. **(B)** Plot of slope vs. the concentration of compound **3f** for the calculation of the inhibition constant *K*_I_. **(C)** Plot of intercept vs. the concentration of compound **3f** for the determination of the inhibition constant *K*_IS_.

For further understanding the interaction mechanism of title compounds with α-glucosidase, compounds **3d**, **3f**, and **3i** were selected to investigate the inhibition mechanism of α-glucosidase through revealing the linkage between enzyme activity and the enzyme concentration in the presence of test compounds (figures for the inhibitory mechanism analysis of compounds **3f** was shown in Figure 2 and figures for the inhibitory mechanism analysis of compounds **3d** and **3i** have been shown in the supporting information). The increasing concentrations of compounds **3d**, **3f**, or **3i** reduced the slope of the lines and the plots of the enzyme activity vs. the enzyme concentration at different concentrations of compounds **3d**, **3f**, or **3i** gave a group of straight lines, which all passed through the origin, indicating that the inhibitor reduces the activity of the enzyme and the inhibition of compounds **3d**, **3f**, or **3i** against α-glucosidase was reversible.

In order to obtain the inhibition kinetics type of compounds **3d**, **3f**, and **3i**, the Lineweaver–Burk plot analysis method was carried out with different concentrations of test compounds and substrates. For compounds **3d**, **3f**, and **3i**, the plots of 1/ν vs. 1/[S] gave a group of straight lines with different slopes that intersected the same point at the second quadrant, indicating that compounds **3d**, **3f**, and **3i** were mixed-type inhibitors. Then, the *K*_I_ values were calculated as 14.96, 33.85, and 22.72 μM, respectively, and the *K*_IS_ values were calculated as 453.85, 58.31, and 24.74 μM, respectively, which are summarized in Table 2. These results showed that compounds **3d**, **3f**, and **3i** could bind with the free enzyme as well as the enzyme–substrate complex of α-glucosidase. In addition, the inhibition types of compounds **3d**, **3f**, and **3i**, different from that of acarbose, are the competitive inhibition type.

**TABLE 2:** Type of inhibition mechanism and *K*_I_ and *K*_IS_ values of compounds **3d**, **3f**, and **3i**.

Compound	Inhibition mechanism	*K*_I_ (μM)	*K*_IS_ (μM)
**3d**	Mixed type	14.96	453.85
**3f**	Mixed type	33.85	58.31
**3i**	Mixed type	22.72	24.74

### 2.5 Molecular Docking Studies

**FIGURE 3:**
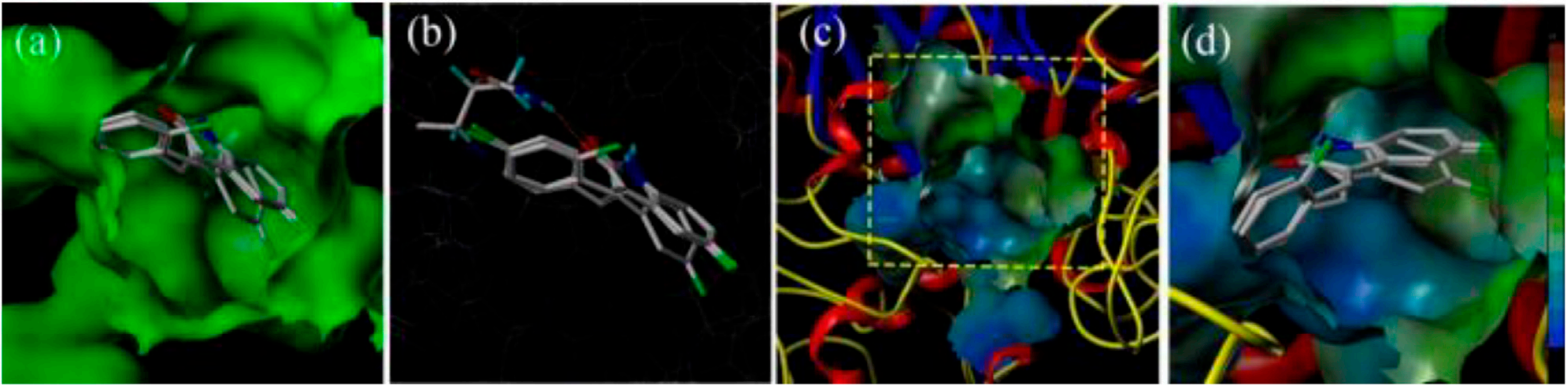
**(A)** The insertion of compounds **3d**, **3f**, and **3i** into the active pocket of α-glucosidase; **(B)** The hydrogen-bond interaction between carbonyl of the compounds (**3d**, **3f**, and **3i**) and α-glucosidase; **(C)** The lipophilic interaction between the compounds (**3d**, **3f**, and **3i**) and α-glucosidase; **(D)** The fluorophenyl as the lipophilic fraction of compounds **3d**, **3f**, and **3i** binding to α-glucosidase.

With the purpose of acquiring a better comprehension of the mutual effects between compounds **3d**, **3f**, and **3i** and α-glucosidase, molecular docking studies were implemented using Sybyl tools. The 3D structures of *S*. *cerevisiae* α-glucosidase (EC 3.2.1.20) are unavailable, and oligo-1, 6-glucosidase from *S. cerevisiae* (PDB: 1UOK) was selected as the target protein. Also, the sequence similarity is about 62.0% and the sequence identity is about 38.0%, as compared with α-glucosidase. As demonstrated in Figure 3A, compounds **3d**, **3f**, and **3i** were well inserted into the active pocket of α-glucosidase, with similar angles and positions. A hydrogen bond between carbonyl of compounds **3d**, **3f**, and **3i** and amino acid sequences of GLN330 was formed to increase the affinity with α-glucosidase (Figure 3B). These similar integrated situations of compounds **3d**, **3f**, and **3i** with α-glucosidase indicated the same inhibition mechanism. In addition, the lipophilic potential interaction between **3d**, **3f**, and **3i** and the active pocket was investigated. As shown in Figure 3C, the active pocket external is more lipophilic than the interior. Then, in Figure 3D, the fluorophenyl as the lipophilic fraction of **3d**, **3f**, and **3i** was close to the lipophilic potential region, while the pyrrole ring as the hydrophilic fraction was near to the hydrophilic region.

## 3 EXPERIMENTAL

### 3.1 Chemicals

α-Glucosidase from *S*. *cerevisiae* (EC 3.2.1.20) and 4-nitrophenyl-β-D-galactopyranoside (*p*-NPG) were supplied by Sigma-Aldrich. All other reagents were of analytical grade. The water used was re-distilled and ion-free.

### 3.2 Instruments

^1^H NMR was recorded by using a NMR spectrometer (DPX-500 MHz) in chloroform-*d* or DMSO-*d*_6_, with chemical shifts (d) given in parts per million (ppm) relative to TMS as internal standard and recorded. Mass spectrometry was determined on a (LCQTM) LC-MS supplied by Thermo Fisher Scientific (Shanghai) Co., Ltd. Melting points were measured on a micro melting point instrument, which was supplied by Shanghai Yidian Physical Optical Instrument Co., Ltd. The absorbance was recorded using a microplate reader supplied by Thermo Fisher Scientific (Shanghai) Co., Ltd.

### 3.3 Synthesis of Compounds 3a ∼ 3v

To a solution of 1 (1.0 mmol, 1.0 equiv.) and **2a ∼ 2v** in 10 ml absolute ethanol was added KOH (6 mmol, 6.0 equiv.), followed by the addition of the corresponding substituted aldehydes. Then, the mixture was stirred at room temperature for 3 h and detected to be complete by TLC. The mixture was adjusted to the pH value between 2.0 and 3.0, followed by the evaporation of ethanol, and extraction with ethyl acetate. The ethyl acetate layer was washed with saturated NaHCO_3_ and brine and then was concentrated under vacuum to give the crude product, subsequently by the recrystallization with ethanol to give compounds **3a ∼ 3v**.

*(Z)*-5-Fluoro-3-(2-Fluorobenzylidene) Indolin-2-One (3a). Orange-yellow crystal; yield 65.0%; m p.: 228.3–230.2°C; ^1^H NMR (500 MHz, DMSO-*d*_6_) *δ* 10.73 (s, 1H), 7.77 (td, *J* = 7.7, 1.7 Hz, 1H), 7.65–7.55 (m, 2H), 7.47–7.36 (m, 2H), 7.13 (td, *J* = 9.0, 2.6 Hz, 1H), 6.94 (dd, *J* = 9.0, 2.6 Hz, 1H), and 6.88 (dd, *J* = 8.5, 4.6 Hz, 1H); HRMS (ESI) calculated for C_15_H_9_F_2_NO [M - H]^−^: m/z = 256.24, found 255.85.

*(Z)*-5-Fluoro-3-(3-Fluorobenzylidene) Indolin-2-One (3b). Orange-yellow crystal; yield 41.2%; m p.: 191.4–192.2°C; ^1^H NMR (500 MHz, chloroform-*d*) *δ* 7.81 (s, 1H), 7.49 (td, *J* = 8.0, 5.7 Hz, 1H), 7.42 (dp, *J* = 7.6, 0.9 Hz, 1H), 7.30 (ddd, *J* = 8.9, 6.8, 2.4 Hz, 2H), 7.20–7.15 (m, 1H), 6.97 (td, *J* = 8.7, 2.5 Hz, 1H), 6.82 (dd, *J* = 8.6, 4.4 Hz, 1H); HRMS (ESI) calculated for C_15_H_9_F_2_NO [M - H]^−^: m/z = 256.24, found 256.09.

*(Z)*-5-Fluoro-3-(4-Fluorobenzylidene) Indolin-2-One (3c). Orange-yellow crystal; yield 55.7%; mp.: 217.0–219.6°C; ^1^H NMR (500 MHz, chloroform-*d*) *δ* 8.10 (s, 1H), 7.82 (s, 1H), 7.64 (dd, *J* = 8.5, 5.4 Hz, 2H), 7.32 (dd, *J* = 9.1, 2.5 Hz, 1H), 7.20 (t, *J* = 8.4 Hz, 2H), 6.96 (td, *J* = 8.8, 2.6 Hz, 1H), and 6.83 (dd, *J* = 8.5, 4.4 Hz, 1H); HRMS (ESI) calculated for C_15_H_9_F_2_NO [M-H]^−^: m/z = 256.24, found 256.17.

*(Z)*-3-(2-Chlorobenzylidene)-5-Fluoroindolin-2-One (3d). Orange-yellow crystal; yield 82.4%; m p.: 219.7–221.9°C; ^1^H NMR (500 MHz, chloroform-*d*) *δ* 8.12 (s, 1H), 7.92 (s, 1H), 7.68 (dd, *J* = 7.5, 1.8 Hz, 1H), 7.54 (dd, *J* = 7.9, 1.4 Hz, 1H), 7.40 (dtd, *J* = 20.5, 7.5, 1.6 Hz, 2H), 7.04 (dd, *J* = 8.9, 2.6 Hz, 1H), 6.94 (td, *J* = 8.8, 2.6 Hz, 1H), and 6.82 (dd, *J* = 8.5, 4.3 Hz, 1H); HRMS (ESI) calculated for C_15_H_9_ClFNO [M-H]^−^: m/z = 272.69, found 271.96.

*(Z)*-3-(3-Chlorobenzylidene)-5-Fluoroindolin-2-One (3e). Orange-yellow crystal; yield 47.8%; mp: 237.4–238.9°C; ^1^H NMR (500 MHz, chloroform-*d*) *δ* 7.97 (s, 1H), 7.79 (s, 1H), 7.58 (dd, *J* = 2.1, 1.1 Hz, 1H), 7.54–7.50 (m, 1H), 7.46–7.43 (m, 2H), 7.25 (d, *J* = 2.5 Hz, 1H), 6.97 (td, *J* = 8.7, 2.6 Hz, 1H), and 6.82 (dd, *J* = 8.5, 4.4 Hz, 1H); HRMS (ESI) calculated for C_15_H_9_ClFNO [M-H]^−^: m/z = 272.69, found 272.10.

*(Z)*-3-(4-Chlorobenzylidene)-5-Fluoroindolin-2-One (3f). Orange-yellow crystal; yield 44.2%; mp: 200.6–202.5°C; ^1^H NMR (500 MHz, chloroform-*d*) *δ* 7.80 (s, 1H), 7.58 (d, *J* = 8.3 Hz, 2H), 7.51–7.45 (m, 2H), 7.30 (dd, *J* = 8.9, 2.5 Hz, 1H), 6.96 (td, *J* = 8.7, 2.6 Hz, 1H), and 6.82(dd, *J* = 8.5, 4.4 Hz, 1H); HRMS (ESI) calculated for C_15_H_9_ClFNO [M-H]^−^: m/z = 272.69, found 272.39.

*(Z)*-3-(2-Bromobenzylidene)-5-Fluoroindolin-2-One (3g). Orange-yellow crystal; yield 69.7%; mp: 194.9–197.1°C; ^1^H NMR (500 MHz, chloroform-*d*) *δ* 7.90–7.82 (m, 1H), 7.73 (dd, *J* = 8.1, 1.1 Hz, 1H), 7.66 (dt, *J* = 7.7, 1.9 Hz, 1H), 7.44 (td, *J* = 7.4, 1.1 Hz, 1H), 7.34 (td, *J* = 7.7, 1.6 Hz, 1H), 7.00 (dd, *J* = 8.9, 2.6 Hz, 1H), and 6.94 (td, *J* = 8.7, 2.6 Hz, 1H); HRMS (ESI) calculated for C_15_H_9_BrFNO [M + Na]^+^: m/z = 340.15, found 340.59.

*(Z)*-3-(3-Bromobenzylidene)-5-Fluoroindolin-2-One (3h). Orange-yellow crystal; yield 70.4%; mp: 222.6–223.8°C; ^1^H NMR (500 MHz, chloroform-*d*) *δ* 8.31 (s, 1H), 7.86–7.67 (m, 2H), 7.66–7.48 (m, 2H), 7.38 (t, *J* = 7.9 Hz, 1H), 7.24 (d, *J* = 2.6 Hz, 1H), 6.97 (td, *J* = 8.7, 2.6 Hz, 1H), and 6.84 (dd, *J* = 8.5, 4.4 Hz, 1H); HRMS (ESI) calculated for C_15_H_9_BrFNO [M + Na]^+^: m/z = 357.15, found 357.97.

*(Z)*-3-(4-Bromobenzylidene)-5-Fluoroindolin-2-One (3i). Orange-yellow crystal; yield 65.7%; mp: 249.3–251.6°C; ^1^H NMR (500 MHz, chloroform-*d*) *δ* 7.77 (s, 1H), 7.67–7.61 (m, 2H), 7.51 (d, *J* = 8.4 Hz, 2H), 7.30 (dd, *J* = 9.0, 2.6 Hz, 1H), 6.96 (td, *J* = 8.7, 2.6 Hz, 1H), and 6.81 (dd, *J* = 8.5, 4.4 Hz, 1H); HRMS (ESI) calculated for C_15_H_9_BrFNO [M + Na]^+^: m/z = 341.15, found 340.97.

*(Z)*-5-Fluoro-3-(2-Methoxybenzylidene) Indolin-2-One (3j). Orange-yellow crystal; yield 52.7%; mp: 227.1–227.8°C; ^1^H NMR (500 MHz, chloroform-*d*) *δ* 8.02 (s, 1H), 7.97 (s, 1H), 7.67 (dd, *J* = 7.7, 1.7 Hz, 1H), 7.49–7.42 (m, 1H), 7.28 (dd, *J* = 9.2, 2.7 Hz, 1H), 7.06 (td, *J* = 7.5, 1.0 Hz, 1H), 7.00 (dd, *J* = 8.3, 1.0 Hz, 1H), 6.91 (td, *J* = 8.8, 2.6 Hz, 1H), 6.80 (dd, *J* = 8.5, 4.4 Hz, 1H), and 3.89 (s, 3H); HRMS (ESI) calculated for C_16_H_12_FNO_2_ [M-H]^−^: m/z = 268.28, found 268.19.

*(Z)*-5-Fluoro-3-(3-Methoxybenzylidene) Indolin-2-One (3k). Orange-yellow crystal; yield 73.3%; mp: 200.1–202.1°C; ^1^H NMR (500 MHz, chloroform-*d*) *δ* 7.86 (s, 1H), 7.45–7.38 (m, 2H), 7.23 (d, *J* = 7.5 Hz, 1H), 7.14 (t, *J* = 2.0 Hz, 1H), 7.04–6.90 (m, 2H), 6.82 (dd, *J* = 8.6, 4.4 Hz, 1H), and 3.86 (s, 3H); HRMS (ESI) calculated for C_16_H_12_FNO_2_ [M-H]^−^: m/z = 268.28, found 268.01.

*(Z)*-5-Fluoro-3-(4-Methoxybenzylidene) Indolin-2-One (3l). Orange-yellow crystal; yield 42.8%; mp: 358.8–359.9°C; ^1^H NMR (500 MHz, chloroform-*d*) *δ* 7.84 (s, 1H), 7.68–7.62 (m, 2H), 7.49 (dd, *J* = 9.3, 2.6 Hz, 1H), 7.04–7.00 (m, 2H), 6.93 (dd, *J* = 8.8, 2.6 Hz, 1H), 6.83 (dt, *J* = 9.0, 3.6 Hz, 1H), and 3.91 (s, 3H); HRMS (ESI) calculated for C_16_H_12_FNO_2_ [M-H]^−^: m/z = 268.28, found 268.11.

*(Z)*-5-Fluoro-3-[2-(Trifluoromethyl) Benzylidene) Indolin-2-One (3m). Orange-yellow crystal; yield 53.1%; mp: 235.2–236.4°C; ^1^H NMR (500 MHz, chloroform-*d*) *δ* 8.33–8.22 (m, 1H), 8.01 (q, *J* = 2.5 Hz, 1H), 7.83 (d, *J* = 7.8 Hz, 1H), 7.70–7.65 (m, 2H), 7.63–7.55 (m, 1H), 6.93 (td, *J* = 8.8, 2.6 Hz, 1H), 6.83 (dd, *J* = 8.5, 4.3 Hz, 1H), and 6.66 (dd, *J* = 8.7, 2.6 Hz, 1H); HRMS (ESI) calculated for C_16_H_9_F_4_NO [M-H]^−^: m/z = 306.25, found 305.91.

*(Z)*-5-Fluoro-3-[3-(Trifluoromethyl) Benzylidene] Indolin-2-One (3n). Orange-yellow crystal; yield 42.9%; mp: 199.7–200.5°C; ^1^H NMR (500 MHz, chloroform-*d*) *δ* 7.87 (s, 1H), 7.85 (s, 1H), 7.81 (d, *J* = 7.7 Hz, 1H), 7.73 (d, *J* = 7.8 Hz, 1H), 7.64 (t, *J* = 7.8 Hz, 1H), 7.18 (dd, *J* = 8.9, 2.6 Hz, 1H), 6.97 (td, *J* = 8.7, 2.6 Hz, 1H), and 6.83 (dd, *J* = 8.6, 4.3 Hz, 1H); HRMS (ESI) calculated for C_16_H_9_F_4_NO [M-H]^−^: m/z = 306.25, found 306.01.

*(Z)*-3-(2,4-Difluorobenzylidene)-5-Fluoroindolin-2-One (3o). Orange-yellow crystal; yield 55.3%; mp: 221.1–224.3°C; ^1^H NMR (500 MHz, chloroform-*d*) *δ* 8.31–8.21 (s, 1H), 8.01 (s, 1H), 7.83 (d, *J* = 7.8 Hz, 1H), 7.68–7.65 (m, 2H), 7.62–7.58 (m, 1H), 6.93 (td, *J* = 8.8, 2.6 Hz, 1H), 6.83 (dd, *J* = 8.5, 4.3 Hz, 1H), and 6.66 (dd, *J* = 8.7, 2.6 Hz, 1H); HRMS (ESI) calculated for C_16_H_9_F_4_NO [M + H]^+^: m/z = 276.23, found 278.01.

*(Z)*-3-(3,4-Difluorobenzylidene)-5-Fluoroindolin-2-One (3p). Orange-yellow crystal; yield 44.6%; mp: 189.2–190.2°C; ^1^H NMR (500 MHz, chloroform-*d*) *δ* 8.99 (dd, *J* = 10.1, 2.7 Hz, 1H), 8.91 (ddd, *J* = 4.6, 2.0, 0.8 Hz, 1H), 7.83 (td, *J* = 7.7, 1.9 Hz, 1H), 7.73 (s, 1H), 7.63 (dd, *J* = 7.7, 1.1 Hz, 1H), 7.36 (ddd, *J* = 7.7, 4.7, 1.1 Hz, 1H), 7.01 (td, *J* = 8.6, 2.7 Hz, 1H), and 6.80 (dd, *J* = 8.5, 4.4 Hz, 1H); HRMS (ESI) calculated for C_15_H_8_F_3_NO [M-H]^−^: m/z = 274.23, found 274.02.

*(Z)*-3-Benzylidene-5-Fluoroindolin-2-One (3q). Orange-yellow crystal; yield 76.3%; mp: 198.9–199.7°C; ^1^H NMR (500 MHz, chloroform-*d*) *δ* 8.93 (s, 1H), 7.90 (s, 1H), 7.67–7.62 (m, 2H), 7.53–7.46 (m, 3H), 7.36 (dd, *J* = 9.1, 2.6 Hz, 1H), 6.94 (td, *J* = 8.7, 2.5 Hz, 1H), and 6.86 (dd, *J* = 8.5, 4.5 Hz, 1H); HRMS (ESI) calculated for C_15_H_8_F_3_NO [M-H]^−^: m/z = 238.25, found 237.99.

*(Z)*-5-Fluoro-3-[4-(Methylthio) Benzylidene] Indolin-2-One (3r). Orange-yellow crystal; yield 41.8%; mp: 246.1–247.1°C; ^1^H NMR (500 MHz, chloroform-*d*) *δ* 8.13 (s, 1H), 7.81 (s, 1H), 7.61–7.55 (m, 2H), 7.44 (dd, *J* = 9.0, 2.6 Hz, 1H), 7.36–7.31 (m, 2H), 6.94 (td, *J* = 8.7, 2.5 Hz, 1H), 6.82 (dd, *J* = 8.5, 4.4 Hz, 1H), and 2.56 (s, 3H); HRMS (ESI) calculated for C_16_H_12_FNOS [M-H]^−^: m/z = 284.34, found 284.13.

*(Z)*-5-Fluoro-3-(Furan-2-ylmethylene) Indolin-2-One (3s). Orange-yellow crystal; yield 72.5%; mp: 237.9–238.8°C; ^1^H NMR (500 MHz, chloroform-*d*) *δ* 7.76 (s, 1H), 7.41 (s, 1H), 7.24–7.16 (m, 2H), 6.88 (td, *J* = 8.8, 2.5 Hz, 1H), 6.82 (dd, *J* = 8.5, 4.2 Hz, 2H), and 6.42 (dt, *J* = 4.0, 2.1 Hz, 1H); HRMS (ESI) calculated for C_16_H_12_FNOS [M-H]^−^: m/z = 228.21, found 227.95.

*(Z)*-5-Fluoro-3-(Thiophen-2-ylmethylene) Indolin-2-One (3t). Orange-yellow crystal; yield 44.6%; mp: 219.0–223.4°C; ^1^H NMR (500 MHz, chloroform-*d*) *δ* 8.01 (dd, *J* = 9.4, 2.5 Hz, 1H), 7.97 (s, 1H), 7.85 (s, 1H), 7.65 (dd, *J* = 23.4, 4.4 Hz, 2H), 7.23 (dd, *J* = 5.1, 3.7 Hz, 1H), 6.99 (td, *J* = 8.7, 2.5 Hz, 1H), and 6.84 (dd, *J* = 8.5, 4.5 Hz, 1H); HRMS (ESI) calculated for C_16_H_12_FNOS [M-H]^−^: m/z = 244.27, found 244.14.

*(Z)*-3-[(1H-Pyrrol-2-yl)methylene]-5-Fluoroindolin-2-One (3u). Orange-yellow crystal; yield 54.4%; mp: 198.9–201.6°C; ^1^H NMR (500 MHz, chloroform-*d*) *δ* 13.30 (s, 1H), 7.76 (s, 1H), 7.41 (s, 1H), 7.22 (s, 1H), 7.19 (dd, *J* = 8.7, 2.5 Hz, 1H), 6.88 (td, *J* = 8.8, 2.5 Hz, 1H), 6.82 (d, *J* = 4.2 Hz, 2H), and 6.42 (dt, *J* = 4.0, 2.1 Hz, 1H); HRMS (ESI) calculated for C_13_H_9_FN_2_O [M-H]^−^: m/z = 227.23, found 227.02.

*(Z)*-5-Fluoro-3-(Pyridin-2-ylmethylene) Indolin-2-One (3v). Orange-yellow crystal; yield 47.9%; mp: 248.5–251.2°C; ^1^H NMR (500 MHz, chloroform-*d*) *δ* 8.99 (dd, *J* = 10.1, 2.7 Hz, 1H), 8.91 (ddd, *J* = 4.6, 2.0, 0.8 Hz, 1H), 7.83 (td, *J* = 7.7, 1.9 Hz, 1H), 7.73 (s, 1H), 7.63 (dd, *J* = 7.7, 1.1 Hz, 1H), 7.36 (ddd, *J* = 7.7, 4.7, 1.1 Hz, 1H), 7.01 (td, *J* = 8.6, 2.7 Hz, 1H), and 6.80 (dd, *J* = 8.5, 4.4 Hz, 1H); HRMS (ESI) calculated for C_13_H_9_FN_2_O [M-H]^−^: m/z = 239.24, found 238.93.

### 3.4 α-Glucosidase Inhibitory Assay

The α-glucosidase inhibition of synthetic compounds was performed as previously reported methods with minor modification (Deng et al., 2022) which is as follows: briefly, 130 μl of phosphate buffer (10 mM, pH 6.8), 10 μl of α-glucosidase (1 U/ml), and 10 μl of test compound solution were added into the wells of a 96-well plate, followed by incubation for 10 min at 37°C. Then, 50 μl of *p*-NPG (1 mM) was added, and the plate was further incubated for 30 min at 37°C. Finally, the absorbance of each well was recorded at 405 nm using a microplate reader. Acarbose was used as the reference. The inhibition of the test compound on α-glucosidase was calculated as follows: inhibition ratio (%) = [(A− B)/A] × 100, where A is the absorbance of blank and B is the absorbance of the test compound. Each concentration was experimented four times in parallel. Half inhibitory concentration (IC_50_) was obtained from the fitting curve of inhibition ratio vs. test compound with different concentrations.

### 3.5 Kinetics Mechanism Analysis

Compounds **3d**, **3f**, and **3i** with much better α-glucosidase inhibitory activity were selected for kinetic analysis. The experiments were performed to investigate the kinetics mechanism of compounds **3d**, **3f**, and **3i** by the previously reported method (Xu et al., 2019). To determine the inhibition mechanism, the final concentrations for **3d** were 0, 40, 50, 60 μM, for 3f were 0, 40, 50, 60 μM, and for **3i** were 0, 30, 40, 50 μM, the final substrate *p*-NPG concentration was 0.25 mM, and the final concentrations for α-glucosidase were 3.75 × 10^–2^, 5.00 × 10^–2^, 6.25 × 10^–2^, and 7.50 × 10^–2^ U/ml. Then, the inhibition rates were measured by the aforementioned method.

To analyze the inhibition type, the final concentrations for **3d** were 0, 40, 50, and 60 μM, for **3f** were 0, 40, 50, and 60 μM, and for **3i** were 0, 30, 40, and 50 μM, the final α-glucosidase concentration was 5.00 × 10^–2^ U/ml, and final concentrations for substrate *p*-NPG concentration were 0.25, 0.50, 0.75, and 1.00 mM. The inhibition rates were obtained by the aforementioned method. The inhibition type on α-glucosidase was analyzed by using Lineweaver–Burk plots of the inverse of velocities (1/v) vs. the inverse of substrate concentration 1/[S]. The *K*_I_ and *K*_IS_ were obtained from the slope and the vertical intercept vs. the inhibitor concentration, respectively.

### 3.6 Molecular Docking

The molecular docking between compounds **3d**, **3f**, and **3i** and α-glucosidase were simulated with Sybyl-2.1.1 (Tripos, Shanghai, China) (Hu et al., 2021). First, compounds **3d**, **3f**, and **3i** were prepared by hydrogenation and energy minimization using the MM2 program. In the energy minimization program, the energy convergence criterion was revised to 0.001 kcal/mol, optimizing the energy gradient that was revised to 2,500 times, and the charge was run with the Gasteiger–Huckle charges method. Next, after being retrieved from the RCSB Protein Database (PDB: 1UOK), the α-glucosidase structure was prepared, followed by the procedure of removing water, termini treatment, adding hydrogens, adding charges with the MMFF94, fixing side chain amides, and staged minimization. The active pocket of α-glucosidase was generated with the automatic mode. Then, the molecular docking between compounds **3d**, **3f**, and **3i** and α-glucosidase were operated in the default format.

## 4 Conclusion

In summary, a series of α-glucosidase inhibitors based on 5-fluoro-2-oxindole have been synthesized and evaluated. Most synthesized compounds presented better potential inhibitory on α-glucosidase than the parent compound. Among them, compounds **3d**, **3f**, and **3i** exhibited much better inhibitory activity with IC_50_ values of 49.89 ± 1.16, 35.83 ± 0.98 and 56.87 ± 0.42 μM, respectively, which were about 10 ∼ 15 folds higher activities than acarbose (IC_50_ = 569.43 ± 43.72 μM) that was used as reference. The kinetics mechanism study revealed that compounds **3d**, **3f**, and **3i** inhibited the α-glucosidase in a reversible and mixed manner. Molecular docking confirmed that compounds could effectively integrate with α-glucosidase. These results indicated that these synthesized compounds could be used as the leading structure in the research and development of α-glucosidase inhibitors for the prevention and treatment of type 2 diabetes.

## Data Availability

The original contributions presented in the study are included in the article/Supplementary Material; further inquiries can be directed to the corresponding authors.
