# Supplementary material for: Synthesis and Biological Evaluation of 5-Fluoro-2-Oxindole Derivatives as Potential α-Glucosidase Inhibitors
Source: Front Chem. 2022 Jun 23;10:928295. doi: 10.3389/fchem.2022.928295 (PMC9261963; doi:10.3389/fchem.2022.928295)
Supplement: Supplementary file 1 [file DataSheet1.docx]

Supplementary Material

**Synthesis and evaluation of 5-fluoro-2-oxindole derivatives as α-glucosidase inhibitors**

Jing Lin^†^, Qi-Ming Liang^†^, Yuan-Na Ye, Di Xiao, Li Lu, Meng-Yue Li, Jian-Ping Li, Yu-Fei Zhang, Zhuang Xiong*, Na Feng*, Chen Li*

School of Biotechnology and Health Sciences, Wuyi University, Jiangmen 529000, P. R. China

†Jing Lin and Qi-Ming Liang contributed equally to this work.

*Corresponding authors: Zhuang Xiong, Na Feng, Chen Li. Email: wyuchemxz@126.com; wyuchemfn@126.com; wyuchemlc@126.com. Tel./fax.: +86 750 3299397

1. IC_50_ of **3d**, **3f**, **3i** and **acarbose** against α-glucosidase ………………………………………..2-3
2. Lineweaver-Burk plots of **acarbose**………………………………….………….......................4
3. Inhibitory mechanism analysis of **3d** and **3i**………………………………………………..…5-6
4. ^1^H NMR of compounds **3a ~ 3v………………………………………………………………7**-17

**Fig. S1** IC_50_ of compound **3d** against α-glucosidase

**Fig. S2** IC_50_ of compound **3f** against α-glucosidase

**Fig. S3** IC_50_ of compound **3i** against α-glucosidase

**Fig. S4** IC_50_ of **acarbose** against α-glucosidase

**Fig.S5** Lineweaver-Burk plots of **acarbose** against α-glucosidase

**Fig.S6** Inhibition mechanism determination of compound **3d** on α-glucosidase

**Fig.S7** Inhibition mechanism determination of compound **3i** on α-glucosidase.

**Fig.8** Lineweaver-Burk plots of compounds **3d** and **3i** on α-glucosidase. Plot of slope vs the concentration of compounds **3d** and **3i** for the calculation of the inhibition constant *K*_I_. Plot of intercept vs the concentration of compounds **3d** and **3i** for the determination of the inhibition constant *K*_IS_.


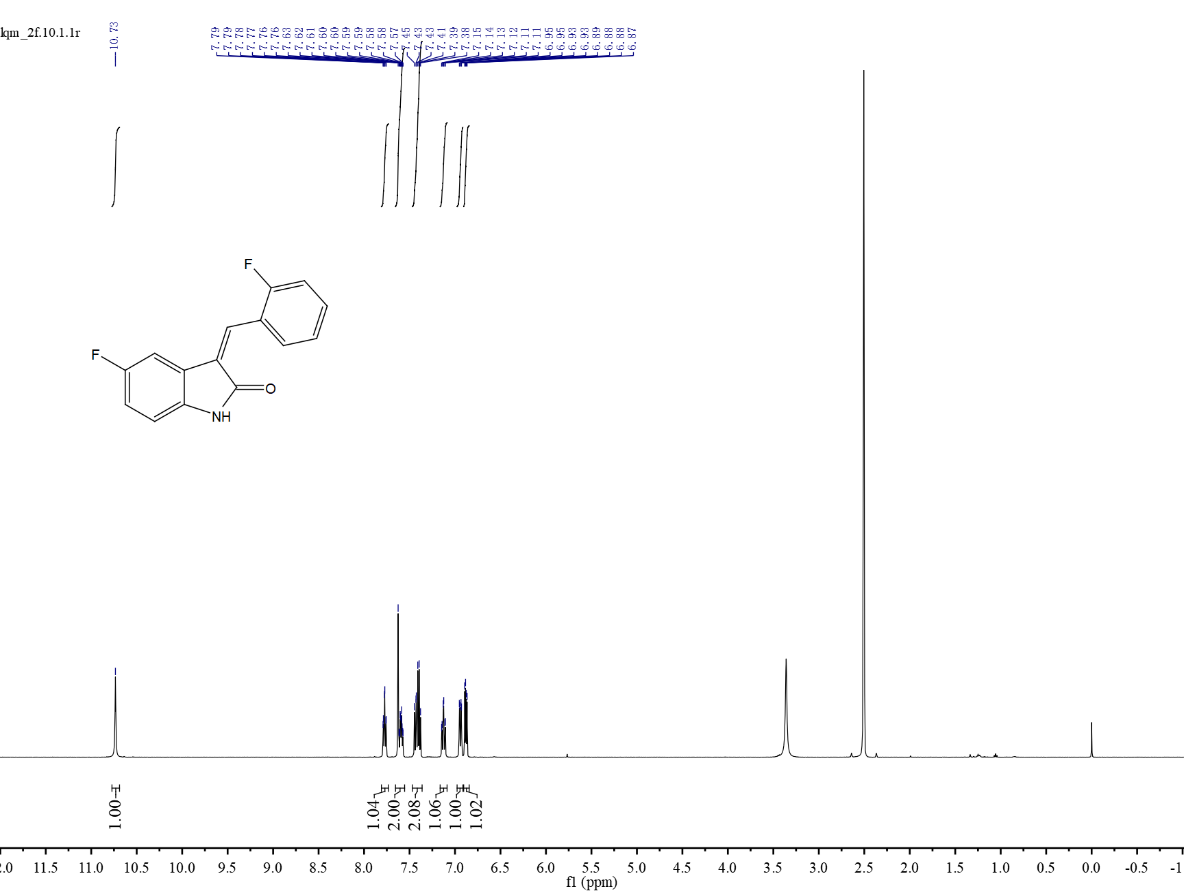


**Fig.S9** ^1^H NMR of compound **3a**

**Fig.S10** ^1^H NMR of compound **3b**

**Fig.S11** ^1^H NMR of compound **3c**

**Fig.S12** ^1^H NMR of compound **3d**

**Fig.S13** ^1^H NMR of compound **3e**

**Fig.S14** ^1^H NMR of compound **3f**

**Fig.S15** ^1^H NMR of compound **3g**

**Fig.S16** ^1^H NMR of compound **3h**

**Fig.S17** ^1^H NMR of compound **3i**

**Fig.S18** ^1^H NMR of compound **3j**

**Fig.S19** ^1^H NMR of compound **3k**

**Fig.S20** ^1^H NMR of compound **3l**

**Fig.S21** ^1^H NMR of compound **3m**

**Fig.S22** ^1^H NMR of compound **3n**

**Fig.S23** ^1^H NMR of compound **3o**

**Fig.S24** ^1^H NMR of compound **3p**

**Fig.S25** ^1^H NMR of compound **3q**

**Fig.S26** ^1^H NMR of compound **3r**

**Fig.S27** ^1^H NMR of compound **3s**

**Fig.S28** ^1^H NMR of compound **3t**

**Fig.S29** ^1^H NMR of compound **3u**

**Fig.S30** ^1^H NMR of compound **3v**
